# Data on microbiological quality assessment of rural drinking water supplies in Tiran County, Isfahan province, Iran

**DOI:** 10.1016/j.dib.2018.04.004

**Published:** 2018-04-06

**Authors:** Khadijeh Jafari, Ali Akbar Mohammadi, Zahra Heidari, Farzaneh Baghal Asghari, Majid Radfard, Mahmood Yousefi, Mahmoud Shams

**Affiliations:** a Students Research Committee, Department of Environmental Health Engineering, Hormozgan University of Medical Sciences, Bandar abbas, Iran; b Department of Environmental Health Engineering, Neyshabur University of Medical Sciences, Neyshabur, Iran; c Bachelor of Environmental Health, Department of Health, Isfahan University of Medical Sciences, Isfahan, Iran; d Department of Environmental Health Engineering, School of Public Health, Tehran University of Medical Sciences, Tehran, Iran; e Department of Environmental Health Engineering, School of Health, Mashhad University of Medical Sciences, Mashhad, Iran; f Torbat Heydariyeh University of Medical Sciences, Torbat Heydariyeh, Iran

**Keywords:** Microbiological quality, Drinking water, Turbidity, pH and chlorine, Tiran

## Abstract

A lack of access to safe drinking water can lead to adverse health effects such as infection, disease, and undesirable aesthetic problems. The current study focused on the investigation of groundwater quality in Tiran's villages (Isfahan province, Iran). To determine essential microbiological quality, water samples were collected from 46 randomly-selected water wells during a one-year period. The parameters of pH and chlorine were measured on-site. Turbidity was measured at 420 nm using a DR5000 spectrophotometer. Microbiological tests including general thermoforms, *Escherichia coli*, and thermophiles were carried out according to the National Iranian Standard Method 3759. Data showed that 1.8% of the villages under study had contaminated water resources. The turbidity values for 94.5% of the resources were within recommended limits (<5NTU). In 20.6% of the samples, the residual free chlorine was in the range of 0 to 0.2 mg/L, 8.79% of samples had values greater than the recommended limits, and18.5% had no free residual chlorine.

**Specifications table**

**Table:** 

**Subject area**	Water microbiology
**More specific subject area**	Microbiology
**Type of data**	Tables, Figure
**How data was acquired**	A total of 552 drinking water samples were collected from 46 villages of the city during a one-year period and on a certain date in standard containers of 500 cc containing sodium phosphate. The remaining free chlorine, pH, and turbidity were recorded by portable kits in the sampling area and measured in the sample vessel. Turbidity was measured at 420 nm using a DR5000 spectrophotometer
**Data format**	Raw, Analyzed
**Experimental factors**	The mentioned parameters above, in abstract section, were analyzed according to the standards for water and wastewater treatment handbook.
**Experimental features**	The levels of physical and chemical parameters were determined.
**Data source location**	Tiran County, Isfahan province, Iran
**Data accessibility**	The data are available whit this article

**Values of data**

• Assurance that water is microbiologically safe for drinking has traditionally been determined by measuring bacterial indicators of water quality, most commonly, total coliforms and fecal coliforms.
• Data analysis showed that between residual chlorine and fecal coliform there is a significant relationship, so that by increasing the amount of residual chlorine, fecal coliform is reduced.
• According to the results, the amount of residual chlorine should be within standard limits (0.2–0.8 mg/L) in order to protect the health of consumers against pathogens such as fecal coliform.

## 1 Data

The data presented here deals with the monitoring of the microbiological quality properties of pH, residual chlorine, and turbidity as shown in Table 1, Table 2. The Pearson correlation between all parameters is shown in Table 3.

**Table 1:** Mean, range and standard deviation of measured microbiological parameters in villages of Tiran city.

**Month**	**Free residual color**		**Coliform**	
	**Mean±SD**	**Range**	**Mean±SD**	**Range**
**September**	0.39±0.37	0–2	1.89±11.18	0–75
**October**	0.45±0.46	0–2	9.45±38.06	0–240
**November**	0.43±0.37	0–2	47.46±204.35	0–1100
**December**	0.39±0.40	0–2	57.39±227.56	0–1100
**January**	0.39±0.35	0–2	9.53±51.01	0–460
**February**	045±0.47	0–2	29.21±164.14	0–1100
**March**	0.36±0.37	0–2	3.56±15.6	0–75
**April**	0.32±0.28	0–1.2	37.35±176.32	0–1100
**May**	0.36±0.49	0–3	3.74±13.89	0–75
**June**	0.52±0.46	0–3	4.48±20.86	0–120
**July**	0.48±0.26	0–1	21.84±102.65	0–523
**August**	0.59±0.51	0–3	104.00±333.92	0–1500

**Table 2:** Mean, range and standard deviation of measured microbiological parameters in villages of Tiran city.

**Month**	**Fecal coliform**		**Turbidity**	
	**Mean±SD**	**Range**	**Mean±SD**	**Range**
**September**	0	0–0	1.63**±**2.05	0.25–8.23
**October**	1.13**±**4.28	0–240	1.41**±**0.89	0.36–4.50
**November**	12.22**±**115.95	0–1100	1.72**±**1.88	0.25–8.23
**December**	13.55**±**116.5	0–1100	1.52**±**1.57	0.25–8.23
**January**	0	0–0	1.68**±**1.87	0.25–8.23
**February**	0.06**±**0.38	0–2.6	1.69**±**1.90	0.25–8.23
**March**	0	0–0	1.46**±**1.54	0.25–7.93
**April**	0	0–0	1.82**±**1.92	0.36–8.23
**May**	0	0–0	1.38**±**1.28	0.25–7.93
**June**	0.07**±**0.45	0–3	1.84**±**1.45	0.25–7.93
**July**	0	0–0	1.65**±**2.05	0.25–8.23
**August**	86.66**±**333.97	0–1500	1.50**±**1.80	0.25–7.93

**Table 3:** Pearson correlation between all parameters.

	**Cl**	**Turbidity**	**Coliform**	**Fecal coliform**
**Cl**	1			
**Turbidity**	0.016	1		
**Coliform**	0.020	−0.023	1	
**Fecal Coliform**	−0.2*	0.042	0.619**	1

** Correlation is significant at the 0.01 level (2-tailed).

* Correlation is significant at the 0.05 level (2-tailed).

## 2 Experimental design, materials and methods

### 2.1 Study area description

**Fig. 1:**
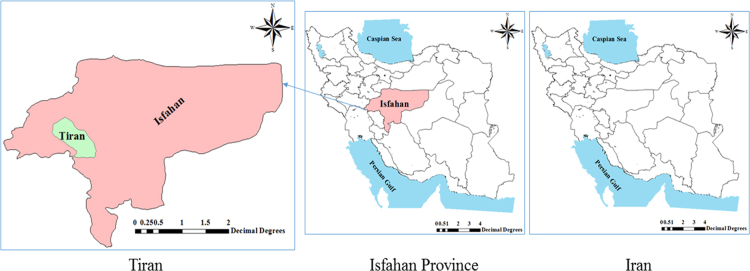
Study area.

The center of the county of Tiran is located at 51°9′6.84″ N and 32°42′12.96″ E and is 1640 m above sea level. The county is located 50 km west of Isfahan (Fig. 1).

### 2.2 Determination of microbiological contamination in drinking water

Samples of drinking water were taken from wells selected in rural areas of Tiran. A total of 552 drinking water samples were collected from 46 villages of the city during a one-year period and on a certain date in standard containers of 500 cc containing sodium phosphate. The remaining free chlorine, pH, and turbidity were recorded by portable kits in the sampling area and measured in the sample vessel. Turbidity was measured at 420 nm using a DR5000 spectrophotometer [1], [2], [3], [4], [5], [6], [7], [8], [9], [10], [11], [12]. Microbiological tests included general thermoforms and *Escherichia coli*. *E. coli* and general thermophilic formulations were carried out according to the National Iranian Standard Method 3759 (multi-tube method, confirmatory and supplementary tests). According to the World Health Organization (WHO), the total number of thermal and *E. coli* forms per 100 milliliters of drinking water should be zero. After collection, all data was analyzed by Excel software, SPSS version 22, and statistical analyses. A level of less than 0.05 was considered significant [1], [2], [13], [14], [15], [16], [17].
