# Supplementary material for: Data on microbiological quality assessment of rural drinking water supplies in Tiran County, Isfahan province, Iran
Source: Data Brief. 2018 Apr 6;18:1122–6. doi: 10.1016/j.dib.2018.04.004 (PMC5996740; doi:10.1016/j.dib.2018.04.004)
Supplement: Supplementary file 2 — Supplementary material [file mmc2.pdf]

EDITORIAL CERTIFICATE LETTER

This document is to certify that the manuscript listed below was edited for proper English language, grammar, punctuation, spelling, and overall style by one of the highly qualified subject-expert native English speaking editors at **NativeEnglishEdit.com**

The substantive content of the article mentioned below remains the full responsibility of the author/authors:

TITLE OF ARTICLE:

DATA ON MICROBIOLOGICAL QUALITY ASSESSMENT OF RURAL DRINKING WATER SUPPLIES IN TIRAN COUNTY, ISFAHAN PROVINCE, IRAN

KHADIJEHJAFARI, ALI AKBAR MOHAMMADI,ZAHRA HEIDARI,FARZANEHBAGHALASGHARI,MAJID RADFARDMAHMOOD YOUSEFI, MAHMOUD SHAMS

EE-2018-1113436 MIRDORAGHIM 5 D

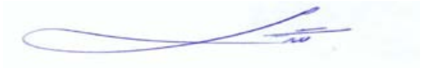

Native English Edit
www.NativeEnglishEdit.com

Documents receiving this certification should be English-ready for publication; however, the author has the ability to accept or reject our suggestions and changes.
This certificate may be verified at:
Native English Edit
www.birminghamresearchpark.co.uk/tenants/native-english-edit
www.NativeEnglishEdit.com
Birmingham Research Park
Edgbaston
Birmingham B15 2SQ
United Kingdom
